# SEEK: a systems biology data and model management platform

**DOI:** 10.1186/s12918-015-0174-y

**Published:** 2015-07-11

**Authors:** Katherine Wolstencroft, Stuart Owen, Olga Krebs, Quyen Nguyen, Natalie J Stanford, Martin Golebiewski, Andreas Weidemann, Meik Bittkowski, Lihua An, David Shockley, Jacky L. Snoep, Wolfgang Mueller, Carole Goble

**Affiliations:** Leiden Institute of Advanced Computer Science Leiden Institute of Advanced Computer Science, Leiden University, 111 Snellius, Niels Bohrweg 1, Leiden, CA 2333 Netherlands; School of Computer Science, University of Manchester, Kilburn Building, Oxford Road, Manchester, M13 9PL UK; Heidelberg Institute for Theoretical Studies, Schloss-Wolfsbrunnenweg, Heidelberg, 35 69118 Germany; School of Chemical Engineering & Analytical Science, The University of Manchester, Oxford Road, Manchester, M13 9PL United Kingdom; Department of Biochemistry, University of Stellenbosch, Private Bag X1, 7602 Matieland, South Africa

**Keywords:** Data management, Model management, Data sharing, Semantic data integration, Metadata standards

## Abstract

**Background:**

Systems biology research typically involves the integration and analysis of heterogeneous data types in order to model and predict biological processes. Researchers therefore require tools and resources to facilitate the sharing and integration of data, and for linking of data to systems biology models.

There are a large number of public repositories for storing biological data of a particular type, for example transcriptomics or proteomics, and there are several model repositories. However, this silo-type storage of data and models is not conducive to systems biology investigations. Interdependencies between multiple omics datasets and between datasets and models are essential. Researchers require an environment that will allow the management and sharing of heterogeneous data and models in the context of the experiments which created them.

**Results:**

The SEEK is a suite of tools to support the management, sharing and exploration of data and models in systems biology. The SEEK platform provides an access-controlled, web-based environment for scientists to share and exchange data and models for day-to-day collaboration and for public dissemination. A plug-in architecture allows the linking of experiments, their protocols, data, models and results in a configurable system that is available 'off the shelf'. Tools to run model simulations, plot experimental data and assist with data annotation and standardisation combine to produce a collection of resources that support analysis as well as sharing. Underlying semantic web resources additionally extract and serve SEEK metadata in RDF (Resource Description Format). SEEK RDF enables rich semantic queries, both within SEEK and between related resources in the web of Linked Open Data.

**Conclusion:**

The SEEK platform has been adopted by many systems biology consortia across Europe. It is a data management environment that has a low barrier of uptake and provides rich resources for collaboration. This paper provides an update on the functions and features of the SEEK software, and describes the use of the SEEK in the SysMO consortium (Systems biology for Micro-organisms), and the VLN (virtual Liver Network), two large systems biology initiatives with different research aims and different scientific communities.

## Background

Research in systems biology is dependent upon the ability to access and integrate heterogeneous data from databases and other published sources as well as on-going experimental investigations. Experimental results and previous work feed into mathematical models, which in turn feed back into future experimental design, creating an iterative cycle between data and models. Scientists therefore need to collaborate through environments that will allow them to exchange data, models and other experimental assets, such as, protocols and standard operating procedures, without exposing pre-publication material to those outside the collaboration. Upon publication, however, encouraging and enabling scientists to share all associated data and models, in standard formats, along with information about how these data are related, is of benefit to the wider scientific community [1].

Appropriate repositories and databases for the public dissemination of systems biology data are currently not available. Although there are large data repositories available for some types of data (e.g. Array Express [2] for transcriptomics, and PRIDE [3] for proteomics), there are few resources that will enable interlinking between different omics datasets and fewer still that enable the management of both data and models. The ISA-TAB standard [4] enables the description of multiple omics datasets and has an associated public repository, but it was not designed for systems biology and therefore does not enable the linking or description of mathematical models.

The ability to interlink heterogeneous data and model collections is essential in systems biology. However, creating such collections is not without cost. Annotation and curation are time-consuming and undervalued processes with few incentives for the individual scientists [5]. Effective data exchange and comparison requires sufficient data annotation. If data or models are not adequately described, they cannot be interlinked or interpreted by others. This is particularly apparent in systems biology, where data heterogeneity means that multiple community metadata standards are required for the annotation of a whole investigation, including data, models and protocols. For example, an investigation involving transcriptomics and proteomics data, with an associated model, would require the transcriptomics data to be MIAME-compliant (Minimum Information about a Microarray Experiment) [6], the proteomics data to be MIAPE-compliant (Minimum Information about a Proteomics Experiment) [7], and the model to be MIRIAM –compliant (Minimum Information Required in the Annotation of Models) [8]. Each standard also recommends the use of specific ontologies as annotation vocabularies. To address these problems, attempts must be made to streamline the annotation process with better tooling, and more incentives must be provided for the scientists to spend time on these activities.

The main challenges for managing data in systems biology are therefore:

1. The large diversity of data
2. The necessity to integrate different types of data
3. The necessity to link data and models (including simulation results and model versioning)
4. Adherence to a broad range of community metadata standards and vocabularies
5. The ability to explore, compare and analyse the data and models produced

The SEEK platform [9] was created to address these challenges, allowing scientists to manage different types of experimental data, and link data with models. The SEEK follows an incremental, standards-compliant development methodology (described fully in Wolstencroft et al. 2011 [9]). It encourages annotation with community ontology terms and standard metadata formats, without enforcing those standards or rigid data structures. In addition, it provides tools for data exploration, annotation and plotting [10]; and for model construction, annotation and simulation [11].

The following sections describe the latest developments in the SEEK software and the associated applications that support systems biology data management. The SEEK is compared to other open source resources available and its use is illustrated in two large systems biology consortia; the European SysMO consortium (Systems biology of Micro-Organisms http://sysmo.net/), and the German Virtual Liver Network http://www.virtual-liver.de/.

## Implementation

The SEEK platform is open-source and built using Ruby on rails. The source code can be downloaded from a BitBucket code repository (https://bitbucket.org/seek4science/seek/wiki/Home), but it is also available as a virtual machine image, in order to allow easy deployment of the whole system (http://www.sysmo-db.org/seek-vm). To date, 30 different instances of the SEEK virtual machine have been downloaded and instantiated for data management support in systems biology projects. These instances support different groups of scientists, ranging from large consortia, like SysMO, to SEEKs that support particular institutes, or individual laboratories.

Following an Agile methodology, new versions and updates of SEEK are released frequently and incrementally. On average, minor upgrades containing bug fixes are released monthly, and major releases are released two or three time a year. Virtual machine packages are also periodically released for key versions that contain significant new features, after a longer period of stabilisation.

**Fig. 1:**
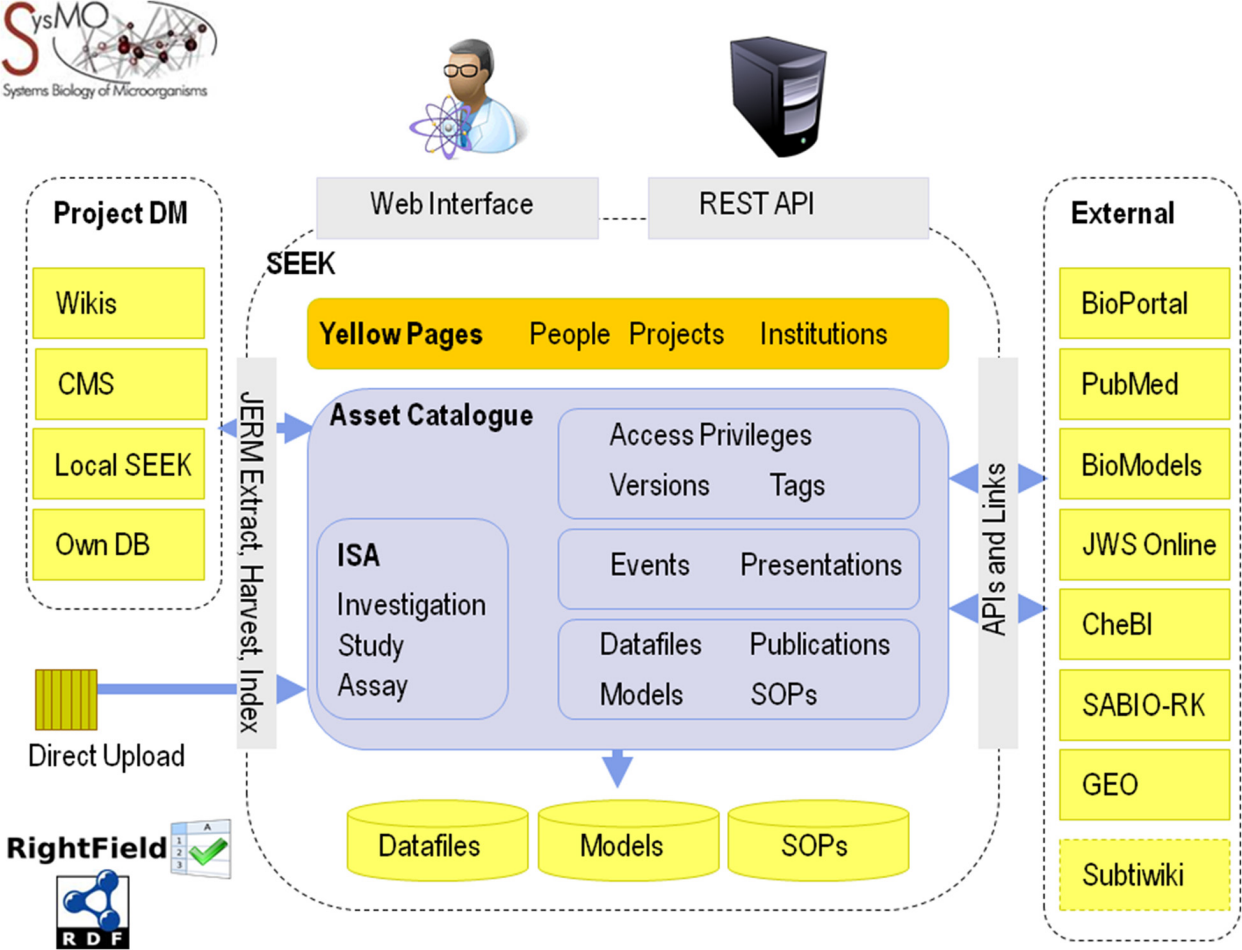
A diagram of the SEEK components. The SEEK is an Assets Catalogue and repository, which links a number of external tools and services and provides a unified, structured interface to all SEEK assets by linking Assays, Studies and Investigations (the ISA Infrastructure)

The SEEK is a collection of components designed to work together, but it is offered as a set of configurable plug-ins to suit individual user requirements (see section *Modularisation* for further details). These components can be combined, customised and included as required for any particular instance. Figure 1 illustrates the different SEEK components and their relationships to each other. The core components include the Assets Catalogue (for storing or linking to data, models and protocols), the *Yellow Pages* directory of consortium members and their expertise, the access control and versioning framework, the Investigations, Studies and Assays (ISA) infrastructure, the harvesting and indexing framework, and the APIs, interfaces and links to external systems biology resources.

**Fig. 2:**
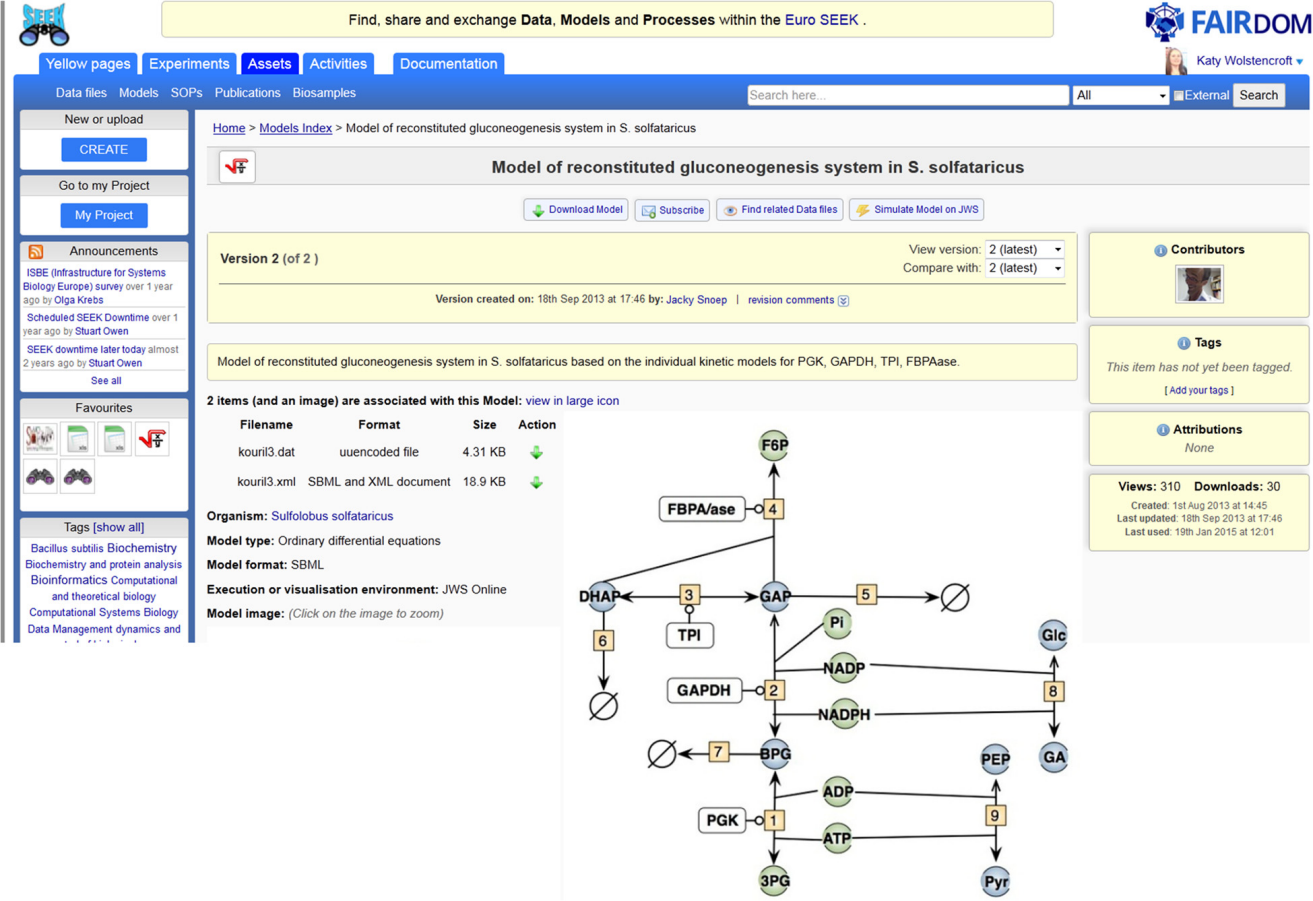
A screenshot of the SysMO-SEEK, which depicts an entry for a model in SBML (Systems biology Markup Language)

The SEEK can be used as either a metadata catalogue or a centralised repository. Data, models and other research assets can be uploaded centrally, or they can be stored remotely and referenced in SEEK with specified minimal metadata. Remote storage includes local content management systems, or other public databases, such as GEO [12] or BioModels [13]. In practice, most SEEK instances operate exclusively as a repository, although the SysMO-SEEK instance (described in Section SysMO-SEEK) originally contained both deposited data and catalogue links. As projects began to reach completion and the emphasis changed from daily collaboration to long-term stewardship and dissemination, all users switched to submitting directly to the central repository. The only external links were to data that had already been deposited in public repositories. The SysMO-SEEK instance provides a 10-year storage guarantee, which enables consortium members to conform to funding agency requirements for data management and provides a persistent URL for published work. Figure 2 shows a screenshot of a model that has been shared and published on SysMO-SEEK.

Since the initial description of the SEEK platform [9], its functionality and utility have been extended. In addition to data management, the SEEK is a platform for exploring and analysing data and models, and an environment for researchers to collaborate with one another. Major additions to the platform include:

- A publishing framework
- A semantic web framework
- Data and model exploration facilities
- Increased modularisation

The following sections describe the new developments in SEEK and the differences in use and configuration between two of the largest adopting consortia.

### Publishing framework

All items uploaded to SEEK have a persistent URL and are linked to the individual scientist who uploaded them. This allows direct references to the items and their creators. These links help to promote credit through data citation as well as rewarding contributions by individuals in a consortium. It is also possible to generate and register a DOI for any SEEK asset that is public and visible (for example https://dx.doi.org/10.15490/seek.1.datafile.1152.2). SEEK instance administrators can add this feature by registering for a DataCite username and password.

In SEEK, individuals are ultimately responsible for registering and sharing their own assets, allowing them to control who has access and when. SEEK assets can be shared publically, shared with the whole consortium, or shared with named individuals and groups. By providing a fine-grained sharing model, the SEEK ensures that scientists remain in control. To further encourage the dissemination of assets after publication, one-click public release is available for related assets. For example, if a scientist makes a model publicly accessible, a report of all data that was used for its construction, simulation, or validation will be displayed. She can select all or a subset of these data to publish along with the model.

For large consortia, whilst it is desirable to incentivise individuals, it is also prudent to release assets only when it benefits the consortium. If further safeguards and administration are required, the roles of *Project Manager*, *Assets Manager* and *Gatekeeper* can be configured. A *Project Manager* is responsible for assigning project membership and administering project-level information, the *Asset Manager* can assume responsibility for managing assets if people leave the consortium, and the *Gatekeeper* can act as a final checkpoint before assets are shared publically.

### Semantic web framework

The majority of data in SEEK are uploaded as Excel spreadsheets and the majority of models are uploaded as SBML (Systems biology Mark-Up Language) files [14]. Uploaded content is indexed using Lucene (http://lucene.apache.org/) and metadata is extracted and stored in RDF (Resource Description Framework, http://www.w3.org/RDF/), which is the W3C standard for data interchange on the web.

Extracting and storing data in RDF allows more complex queries to be formulated either across an individual SEEK instance, or potentially across multiple federated resources via the Linked Data cloud (http://lod-cloud.net/).

Individual SEEK instances can be configured with or without RDF support. For those that extract and store data in RDF, a SPARQL endpoint (http://www.w3.org/TR/sparql11-overview/)for querying the RDF can be exposed. An example of a SEEK SPARQL endpoint populated from SysMO-SEEK metadata, and a collection of example queries, can be found at https://wiki.sysmo-db.org/seek/sparql-examples. Documentation for setting up the RDF triplestore and configuring the RDF framework is available at:

http://seek4science.org/sites/default/files/seekdocs-0.22.0/doc/SETTING-UP-VIRTUOSO.html

Currently, this is an *advanced* user feature. We do not anticipate that many SEEK users will search SEEK contents directly from the SPARQL endpoint. Instead, the SPARQL endpoint will be used in visualisation and analysis applications, in order to make complex querying more accessible. A detailed description of the semantic web framework and the types of complex queries requested and designed by SEEK users is available in Wolstencroft et al. 2013 [15]. This paper also compares and evaluates Lucene/solr querying against RDF/SPARQL.

A major advantage of serving SEEK metadata as RDF is that it enables greater interoperation with other related resources, such as ArrayExpress [2], ChEBI [16], or the collective content of the EBI RDF platform [17], or Bio2RDF [18]. A major finding in Wolstencroft et al. 2013 was that Lucene/solr and RDF/SPARQL queries performed equally well on many user questions, with the exception of those questions that involved the inclusion of external data or ontology sources. The RDF/SPARQL interface can therefore extend the capabilities of SEEK. The recent production of an RDF representation of ISA-TAB also provides further possibilities for interoperability between SEEK and other ISA-structured resources [19].

The JERM (Just Enough Results Model) is the underlying data model in SEEK [15]. Like MIAME or MIRIAM, it is designed to describe the *minimum information*, which means it describes the basic set of metadata elements required in order to find and interpret SEEK data. The JERM ontology (available from the BioPortal [20], http://bioportal.bioontology.org/ontologies/1488) formalises these relationships. RDF extracted from SEEK conforms to this ontology model, allowing complex queries and inferences over the data.

The JERM describes the relationships between the SEEK assets and the content of those assets. For example, for each dataset uploaded to SEEK, the JERM describes what type of experiment it was, what was measured, and what the values in the dataset mean. The JERM captures the core elements of metadata shared by existing minimum information guidelines, allowing users to comply with these standards as well as capturing the information required for linking in SEEK. Where different types of experiments require the same metadata elements, datasets can be aggregated. There is no requirement to homogenise content that is unique to any one experiment type.

This flexibility is a major advantage of the RDF approach and contrasts with relational database approaches that would require changes to the underlying data model in order to accommodate new experimental data types. SEEK users are therefore able to add new data types as their experimental approaches expand. To assist users in producing data in JERM-compliant formats, spreadsheet templates are provided that encapsulate the JERM and other minimum metadata standards. These templates have been augmented with ontology term selection functions, using the RightField [10] semantic annotation tool (also developed in this project). RightField enables lists of ontology terms (from the web locations, like BioPortal, or from local files) to be embedded into specific spreadsheet cells. As scientists annotate their data, they can select appropriate ontology terms from simple drop-down lists, without requiring any knowledge of the ontology or its structure.

SEEK users can select from a range of JERM-compliant spreadsheet templates from the help section in the SEEK platform (e.g. the SysMO template collection is available from https://seek.sysmo-db.org/help/templates). The main advantage of this approach is that the majority of data is already collected via spreadsheets. To comply with the standards recommended by SEEK, users do not need to make large modifications to their current working practices and they do not need to use new applications.

For models, the annotation tool that is integrated in the JWS simulator follows the MIRIAM guidelines in annotating SBML models, using the Semantic SBML Web Services [21]. The MIRIAM specification requires species and reactions in models to be annotated with official identifiers and recommends the use of terms from community ontologies to describe model elements. MIRIAM annotation ultimately improves interlinking with experimental datasets because components in models are annotated with the same biological identifiers as the datasets.

### Data and model exploration

Data and models in systems biology investigations are inherently interlinked. The SEEK provides tools to assist users in understanding and exploring those links and visualising data and models.

**Fig. 3:**
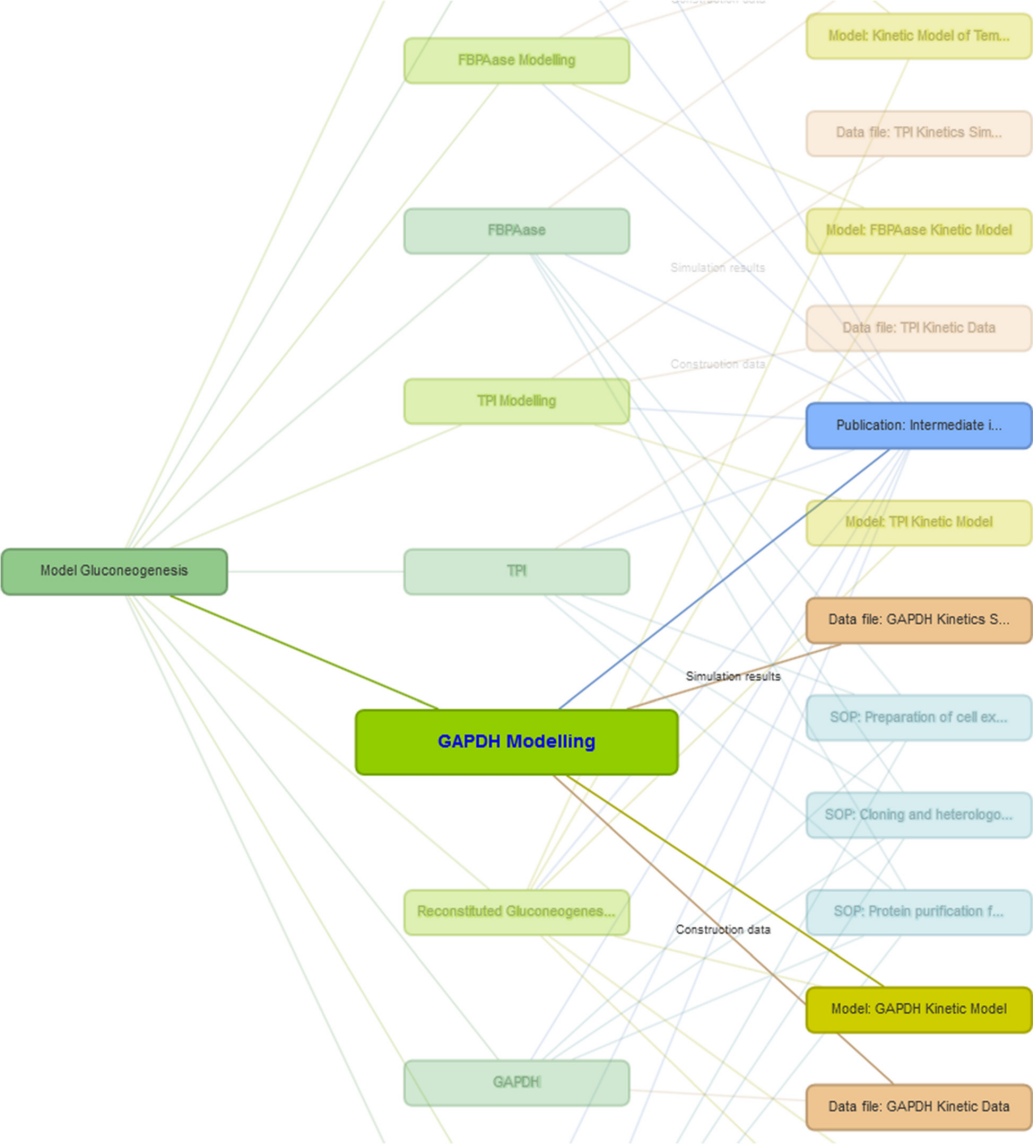
The Investigation, Study Assay view of the Model Gluconeogenesis Study in the SysMO SEEK (https://seek.sysmo-db.org/studies/112). The figure shows which assays are associated with the study and which experimental assets are, in turn, associated with the assays. The GAPDH assay is highlighted, showing it is associated with a model, a publication and two data files; one which was used for model construction and one which shows results of a model simulation

The ISA structure (Investigations, Studies and Assays) is central to the organisation and visualisation of all assets in the SEEK. An ISA tree-view describes which Assays belong to which Studies and which Studies belong to which Investigations. Assays in the ISA-TAB specification refer only to experimental assays. In SEEK, however we extend this description to encapsulate modelling analyses and bioinformatics analyses. Conceptually, they are the same, but they have different sets of metadata descriptions. For example, a modelling assay should be defined by the biological problem being addressed by the model and the modelling framework being used. This extension allows researchers to associate modelling and experimental activities with one another, giving a complete overview of all work associated with a particular investigation. An example can be seen in Fig. 3.

There are a number of different options for visualising and analysing the content of the data and models in SEEK. For example, the *Explore Data* application allows users to view the contents of spreadsheets without downloading them. This view can also be used to add further annotation to the data, or to select data values from the spreadsheets and plot them. Plots can be saved as annotations to the data sheet, or they can be exported and saved separately.

For visualising and exploring models, the JWS Online simulation environment is embedded in SEEK. Through JWS Online, users can view their models in SBGN (Systems biology Graphical Notation) [22], simulate them with the data and parameters provided, or simulate them with alternative values, which could be from other SEEK files, or from elsewhere. Models that are uploaded with a Cytoscape web compatible file (XGMML), can also be visualised using a Cytoscape plugin [23].

The current MIRIAM standard for model annotation enables the identification of model species and parameters, but recording the source of parameter values is not a minimum requirement and may therefore be omitted in many cases. Experimental data containing these values may be included in a table in a publication, but is not readily accessible from model repositories and not amenable to computational processing. The SEEK provides a common interface to display and preserve links between data and models, allowing modellers to record which data was used for model construction and which for validation. The data itself is linked to all the contextual information required for others to interpret the results and determine the validity of using those values in the model. For example, data can be linked to the standard operating procedures and protocols that were followed during its creation. Figure 3 shows an example of intra-experimental connections. Data that is associated with a model can either be annotated as *construction, validation, or simulation*.

By sharing and linking data and models, and allowing model simulations, the SEEK promotes the reuse of existing resources. Users can simulate models with the original data, or run new simulations with other data in SEEK. Users can also search and access external modelling repositories, such as BioModels, in order to further promote reuse.

On-going work with the SED-ML model simulation format [24] enables SEEK users to record these *in silico* simulation results alongside experimental values, or directly compare simulation data with experimental results. The JWS simulator in SEEK allows the export of any model simulation as a SED-ML archive. Through a collaboration with the University of Rostok, BiVeS (the BioModel Version Control System, https://sems.uni-rostock.de/bives/) has recently been integrated with SEEK. This supports the comparison of SBML models, to detect differences at the XML level, and provide a summary of the differences along with a graphical representation. To use this feature, users must have an account in SEEK. For a demonstration, guest users can log into a demo version of SEEK (https://demo.sysmo-db.org/models/33).

### Modularisation

SEEK is developed using a modular approach, making it easy to add and remove given features and behaviours. There are configuration points for turning certain features on and off, supporting customisation when setting up a new installation of SEEK for certain purposes. These are defined in the document:

https://github.com/seek4science/seek/blob/master/lib/seek/config_setting_attributes.yml

An example of the configuration options used in the SysMO SEEK can be found here:

https://github.com/seek4science/seek/blob/master/config/initializers/seek_configuration.rb-openseek

Developers wanting to adapt SEEK can leverage the modular nature of SEEK to more easily integrate new features or modify existing features. We leverage the Rails built in Plugin and Gem system. All the plugins and gems we use are listed in our gemfile: https://github.com/seek4science/seek/blob/master/Gemfile (not all created by and for SEEK).

SEEK is also being updated to use JQuery and the Bootstrap framework to make it easier to theme and customise the user interface following modern conventions.

### Programmatic access

SEEK provides a RestFul API, which is currently read-only. Any resource in SEEK can also be represented as XML, by requesting it in this format instead of HTML through content-negotiation. This is also possible by putting a .xml at the end of the URL, for example https://demo.sysmo-db.org/investigations/2.xml. This XML is backed and validated against an XSD schema, available at https://github.com/seek4science/seek/blob/master/public/2010/xml/rest/schema-v1.xsd.

As well as XML, SEEK also provides an RDF representation, for example https://demo.sysmo-db.org/investigations/2.rdf.

Future plans include updating the Restful API to support JSON, and also to add write access for some key actions such as adding a data file, or defining an assay.

## Related work

Many Life Science disciplines face the challenge of managing large-scale omics data. As a consequence, a number of open resources have been produced to manage particular types of data (e.g. the Metabolights repository for metabolomics data [25]). However, there are fewer resources dedicated to managing data spanning multiple omics types, or managing the link between datasets and models.

The ISA Tools suite [4] and openBIS [26] address cross-omics data exchange, and DIPSBC [27] and the Bioinformatics Resource Manager [28] directly address systems biology data exchange. Wruck et al. (2012) [1] compares the differences between related systems in depth.

**The ISA tools** suite provides java-based desktop clients for designing and managing data in the ISA-TAB format. Users can design metadata descriptions of their experiments, incorporating ontology term selection, in a "spreadsheet-like" interface. The accompanying BII database provides a repository for sharing data in this format, but it is designed for post-publication data exchange, since it does not provide access control.

The ISA-Tab specification organises experimental descriptions into their Investigations, Studies and Assays, and is used to describe the relationships between different omics experiments. It is becoming a de-facto standard, with many tools (Including SEEK) adopting the ISA-Tab format for metadata exchange [29]. The focus of ISA tools, however, is purely on experimental data. In SEEK, the ISA structure is used to organise and link related experiments, but it has been extended to incorporate the relationships between the omics data and models. ISA tools and SEEK have similar and complimentary approaches to multi omics data exchange and ISA-RDF will enable easier integration between SEEK and other ISA resources in the future.

**openBIS** (Open Biological Information System), developed at ETH Zürich in the SyBIT project, is designed to streamline raw data transfer from instruments into analysis workflows. The openBIS system caters for high content screening, proteomics, metabolomics and deep sequencing data sets. It has a configurable metadata and annotation system and the software is extensible. The focus of openBIS on the primary data acquisition processes makes their approach complementary to that of SEEK, which largely manages derived and processed data. openBIS and SEEK are consequently being integrated in the FAIRDOM project (http://fair-dom.org/), which aims to establish a support and service network for European systems biology.

**DIPSBC** - (Data Integration Platform for systems biology Collaborations) was designed specifically for managing systems biology data. Like SEEK, it is compliant with existing community metadata standards, but it accepts and parses XML representations of the data, rather than spreadsheets. For certain types of experimental machines that produce XML directly (e.g. mass spectrometry), this is a straight-forward process. However, for other types of data, the requirement for generating XML representations of data may be a barrier to adoption. Data uploaded to DIPSBC is indexed and searched using Solr/Lucene in the same way as in earlier versions of SEEK, and a Foswiki interface allows users to create, share and manage versions of pages and resources as required. Unlike SEEK, however, resources do not receive a persistent URL for referencing from publications, and individual researchers are not associated with the data they upload.

**The Bioinformatics Resource Manager (BRM)** is a data warehouse system that imports data from a range of public sources (such as KEGG, NCBI and the Gene ontology), and allows users to combine this public data with local data files. Data is incorporated or exported using wizards in the client. Like SEEK, local data can be uploaded centrally or stored locally. Although the BRM is designed for systems biology, there is currently no support for managing or incorporating models, but it is possible to perform analyses of the data via integration with the gaggle infrastructure [30].

**LabKey** [31] is an open source, web-based data management system. It has been specifically designed to manage proteomics, flow cytometry and observational study management data, but it can be extended to manage other data types. It has a relational database backend from which users can import and export spreadsheet data and it has a role-based system for managing access control. LabKey is exclusively for data management, so it has no provision for managing models. It does, however, include a collection of tools for exploring and analysing data, including integration with the R scripting environment.

**KBase** is an open-source software platform designed to facilitate sharing of data and tools for the generation and sharing of hypotheses in systems biology. It provides tools for annotating and simulating heterogeneous datasets, access to data from different organisms as a single data model, community sharing of resources including data and training material. It is not a repository, but relies on linking to databases.

## Results and discussion

### SEEK in use

The SEEK platform was originally developed to support scientists from the SysMO consortium (Systems biology of Micro Organisms http://www.sysmo.net), which is a trans-European initiative of over 300 scientists in over 100 research groups. It has subsequently been adopted by a number of other consortia, in systems biology, for example, the Virtual Liver project; or related disciplines, for example, the BioVel SEEK for biodiversity data management. As the user community of SEEK expanded, new features and functionalities were required. In this section we describe a usage case study from the original SysMO-SEEK platform and then explore the differences in the Virtual Liver research community that lead to new features and developments in the Virtual Liver instance of SEEK.

### The SysMO-SEEK

SysMO is a trans-European initiative of over 300 scientists in over 100 research groups. To date, the SysMO-SEEK (https://seek.sysmo-db.org/) contains over 2000 research assets (i.e. data files, models and protocols) that describe the work of the consortium and the relationships between their data files and models. Many SysMO-SEEK research assets relate to published work and are publicly available. In the past 6 months, there have been more than 3000 unique downloads of data files and models, which demonstrates the reusability of the contents and the value of sharing and publishing through SEEK. Other assets are yet to be published and are therefore only accessible to the SysMO consortium, but we anticipate more will be released as work is finalised and published.

The SysMO consortium was a 6-year initiative to pool systems biology research and know-how across the European research community. It is representative of a typical large-scale systems biology initiative. Work was divided into projects, whose members were distributed and multi-disciplinary. Models and data were produced in each project, using different experimental and modelling techniques, including multiple omics investigations (e.g. transcriptomics, proteomics and metabolomics).

The research themes throughout the consortium were diverse, but the techniques and approaches for tackling the iterative cycle between experimentation and modelling could be propagated and shared, promoting best-practice within and beyond the consortium. Biological topics in SysMO range from fundamental research with model organisms (i.e. yeast and *E.coli*), to industrial microbe production in biofuels (e.g. *Clostridium acetobutylicum)* and human microbial infection (e.g. *Streptococcus pneumoniae)*.

The following section describes a typical SysMO investigation. It describes experimental data and mathematical models, and their relationships to each other. More importantly, it describes the reuse of data from the literature and the reuse of detailed kinetic models in a modular modelling approach.

### SysMO-SEEK: a case study of data and models interaction

Investigation 51 in the SysMO SEEK describes a series of experiments on the central carbon metabolism of the thermophilic bacteria, *Sulfolobus solfataricus* (https://seek.sysmo-db.org/investigations/51). It focuses on the specific adaptations in regulation for high temperature conditions and the effects on stability for intermediates in the gluconeogenic pathway [32]. The investigation encompasses mathematical modelling and experimental biology. All data files associated with the investigation are annotated using RightField-enabled templates. The kinetic constants and rate equations are included in the SABIO-RK database [33], and models are provided in MIRIAM-compliant SBML.

Four enzymes from the gluconeogenic pathway were purified and kinetically characterised and the stability of pathway intermediates was quantified by *in vitro* reconstitution of the enzymes. A mathematical model was constructed on the basis of these data, and the model was able to quantitatively predict the system fluxes and metabolite concentrations. The study demonstrated that intermediate instability can significantly affect pathway efficiency.

The Investigation contains a study involving the creation of a gluconeogenesis model. Kinetic characterisation experiments (assays) were conducted on the four most temperature sensitive intermediates in the gluconeogenic pathway and these data were used to create individual models of their kinetics. By combining these models, a detailed model for the pathway was produced. In a second study assessing carbon loss at high temperatures, the model was used to predict the dynamics of an *in vitro* reconstituted system without further fitting. Experimental data validated the results of simulations. Figure 3 shows the ISA-view of the Model Gluconeogenesis study. It shows how data files, models, and Standard Operating Procedures are inter-related and provides a simple framework for navigating experimental output.

### The virtual liver SEEK: New challenges from multi-scale systems biology

The Virtual Liver Network (VLN - http://www.virtual-liver.de) adopted SEEK as a data management platform due to the success of the SysMO-SEEK. The Virtual Liver Network is a national research initiative in systems biology funded by the German Federal Ministry for Education and Research (BMBF). The largest difference between the work in SysMO and VLN is that VLN data and models are derived from eukaryotes and therefore span multiple scales of biology. This has an impact on the size of data and the strategies for managing and visualising the connections between scales.

#### Enhanced uploading support

Within the VLN, scientists have to exchange much larger data files than in SysMO (e.g. histological images of liver slices can be several GB in size. The file size is only limited by the size of the partition of the SEEK server instance where the file is stored; the biggest file size tested was 27 GB). Upload and exchange of these files is not supported by all major web browsers. To this end and to simplify data upload in general, a Java Webstart application was developed. One or more data files can be simply transferred by 'Drag-and-Drop' operations from the file operating system to the Java application. Additionally, by 'dropping' a folder, all files of this folder as well as any sub-folders are packed into a zip-archive. The file attributes, including the file location, are stored in an intermediate queue and the files are then consecutively send by the tool to a SEEK central data repository using the HTTP post protocol. The files are intermediately stored in a queue and automatically sent by the tool to a SEEK central data repository. Finally, both sender and recipient are notified by email upon file upload. The notification includes links to SEEK pages where the data are accessible. By default, the data are only visible to the sender and the recipient on the SEEK server, but sharing rights can be granted to other users or groups on the corresponding SEEK data upload page.

#### Biological scale-slider

In the Virtual Liver SEEK, users can assign scales to assets, ranging from whole organisms, through organs to cells, and search or filter the content by scales. The scales are visualised and selected by the use of an interactive scale-slider image depicting the liver and its components (http://seek.virtual-liver.de).

### Managing and sharing biological samples

In SysMO, information about biological samples and treatments were collected as part of the metadata pertaining to an experiment. The generation of samples was typically tightly coupled to either an individual Assay or a Study, so it was considered part of that experiment. In the Virtual Liver, the treatment of specimens and the generation of samples can be completely de-coupled. For instance, one research group obtains livers of mice that have undergone certain treatments and distributes slices of these livers to different collaborators within the Virtual Liver Network. All partners need to cross-link to the corresponding results from many different assays based on the same source material. In such a scenario, the sample and treatment data should be uploaded independently from, and usually before, the experimental data. Manual data input would be time-consuming and error-prone as some laboratories produce hundreds of samples with different kinds of treatments, which are then used by different groups in the VLN. Therefore, the Biosamples database and user interface in the VLN-SEEK allows for the de-coupled processing and bulk upload of sample and specimen information.

In addition to parsing and extracting sample information from JERM-compliant templates, the VLN SEEK has developed a general spreadsheet parser for managing legacy data that does not conform to recommended formats. For a given custom spreadsheet format the VLN parser allows users to define which columns contain what information. Once mapped, the data can be transformed into the VLN SEEK internal metadata representation.

VLN SEEK users were able to bulk import hundreds of sample descriptions together with information about related specimens and treatments. Besides defining a simple mapping from columns to data items within VLN SEEK, more complex transformations were required in cases where multiple data items were aggregated into single columns, or where information was only available implicitly in column headers. For missing data, the definition of default values was required.

The creation of a new spreadsheet mapping is currently a task shared by a biological expert, who defines the semantics of a mapping, and a software developer, who implements this mapping in the appropriate way. In future, this process will be simplified by using data reshaping tools, such as Open Refine (formerly known as Google Refine), as an interface to define mappings without the help of an intermediate software developer. In conjunction with RightField, mapping legacy data to a common format, or providing standards-based templates, both contribute to the uniform and standard collection of systems biology data.

## Conclusion

The suite of resources available through the SEEK platform provides an out-of-the-box solution for managing and sharing data and models in systems biology. SEEK supports scientists in individual laboratories, institutes or consortia and allows users to select customised sets of tools for their own specific needs.

One of the main advantages of the approach adopted by SEEK is that it encapsulates the process of standardising and annotating data into commonly used tools, and shields the complexities of metadata models and ontologies from the users. Researchers need to make only small modifications to their current working practices to comply with SEEK recommendations, so the barrier to entry is low. Another major advantage of SEEK is the consideration of the research asset life cycle. Researchers will generally only share data and models publicly when they wish to use them in support of a publication, but they may wish to share with a variety of collaborators beforehand. By providing a fine-grained access control system, and an easy mechanism to make data public at a later date, SEEK therefore supports collaborative work in consortia or distributed projects as well as acting as a repository for disseminating published work.

Many researchers recognise the importance of accessing and using data in the public domain, especially in the multidisciplinary field of systems biology, but giving researchers enough incentives to contribute to this pool is more difficult. SEEK is a step towards changing this culture. By combining the use of specialised tools to make annotation and standardisation more straight forward, with the added reward of association and ownership of data, SEEK provides greater incentives for data sharing. The FAIRDOM project will harness the emerging sharing culture by implementing a European-wide FAIRDOMHub platform. FAIRDOMHub will consolidate existing SEEK resources, integrate openBIS and SEEK platforms, and provide an environment for consortia and independent research groups to share research assets.

In systems biology, the interaction and iterative cycle between experimental work and mathematical modelling are central to the discipline. Managing systems biology projects must therefore involve the management of both data *and* models and it must manage their integration. There are a number of open source applications available for storing heterogeneous systems biology data and a number that tackle the storing, sharing and simulation of models, but SEEK brings these elements together to provide a practical open-source environment for collaboration.

## Availability and requirements

**Project name:** SEEK

**Project home page:**http://www.seek4science.org

**Operating system(s):** Linux is the preferred environment, and Mac OS X is also supported. Microsoft Windows is not supported. A VirtualBox based virtual machine is also available.

**Programming language:** Ruby (version 2.1.x), Java 6 or 7 (including OpenJDK)

**Other requirements:** Ruby on Rails environment – version 3.2.x

**License:** New BSD

**Any restrictions to use by non-academics:** no

## Abbreviations

RDF: Resource Description Framework
SysMO: Systems biology for Micro-Organisms
VLN: Virtual Liver Network
ISA: Investigations, Studies, Assays
ISA-TAB: Investigations, Studies, Assays, Tabular format
MIAME: Minimum Information About a Microarray Experiment
MIAPE: Minimum Information About a Proteomics Experiment
MIRIAM: Minimum Information Required in the Annotation of Models
JERM: Just Enough Results Model
SBML: Systems biology Markup Language
SBGN: Systems biology Graphical Notation
SED-ML: Simulation Experiment Description Markup Language
